# The impact of virtual reality simulation training on operative performance in laparoscopic cholecystectomy: meta-analysis of randomized clinical trials

**DOI:** 10.1093/bjsopen/zrac086

**Published:** 2022-07-18

**Authors:** Gemma Humm, Helen Mohan, Christina Fleming, Rhiannon Harries, Christopher Wood, Khaled Dawas, Danail Stoyanov, Laurence B Lovat

**Affiliations:** Wellcome/Engineering and Physical Sciences Research Council Centre for Interventional and Surgical Sciences. University College London, London, UK; UCL Division of Surgery and Interventional Science, University College London, London, UK; Peter MacCallum Cancer Centre, Melbourne, Victoria, Australia; Department of General and Colorectal Surgery, University Hospital Limerick, Limerick, Ireland; Department of General Surgery, Swansea Bay University Health Board, Swansea, UK; Department of General Surgery, University College London Hospitals NHS Foundation Trust, London, UK; UCL Division of Surgery and Interventional Science, University College London, London, UK; Wellcome/Engineering and Physical Sciences Research Council Centre for Interventional and Surgical Sciences. University College London, London, UK; Wellcome/Engineering and Physical Sciences Research Council Centre for Interventional and Surgical Sciences. University College London, London, UK; UCL Division of Surgery and Interventional Science, University College London, London, UK

## Abstract

**Background:**

Simulation training can improve the learning curve of surgical trainees. This research aimed to systematically review randomized clinical trials (RCT) evaluating the performance of junior surgical trainees following virtual reality training (VRT) and other training methods in laparoscopic cholecystectomy.

**Methods:**

MEDLINE (PubMed), Embase (Ovid SP), Web of Science, Scopus and LILACS were searched for trials randomizing participants to VRT or no additional training (NAT) or simulation training (ST). Outcomes of interest were the reported performance using global rating scores (GRS), the Objective Structured Assessment of Technical Skill (OSATS) and Global Operative Assessment of Laparoscopic Skills (GOALS), error counts and time to completion of task during laparoscopic cholecystectomy on either porcine models or humans. Study quality was assessed using the Cochrane Risk of Bias Tool. PROSPERO ID: CRD42020208499.

**Results:**

A total of 351 titles/abstracts were screened and 96 full texts were reviewed. Eighteen RCT were included and 15 manuscripts had data available for meta-analysis. Thirteen studies compared VRT and NAT, and 4 studies compared VRT and ST. One study compared VRT with NAT and ST and reported GRS only. Meta-analysis showed OSATS score (mean difference (MD) 6.22, 95%CI 3.81 to 8.36, *P* < 0.001) and time to completion of task (MD −8.35 min, 95%CI 13.10 to 3.60, *P* = <0.001) significantly improved after VRT compared with NAT. No significant difference was found in GOALS score. No significant differences were found between VRT and ST groups. Intraoperative errors were reported as reduced in VRT groups compared with NAT but were not suitable for meta-analysis.

**Conclusion:**

Meta-analysis suggests that performance measured by OSATS and time to completion of task is improved with VRT compared with NAT for junior trainee in laparoscopic cholecystectomy. However, conclusions are limited by methodological heterogeneity and more research is needed to quantify the potential benefit to surgical training.

## Teaser

A systematic review and meta-analysis of randomized clinical trials investigating the impact of virtual reality training on operating performance in laparoscopic cholecystectomy was undertaken. Results show that virtual reality training could improve performance and reduce operating time compared with no additional training in laparoscopic cholecystectomy for junior surgical trainees. VR training has advantages over no additional training and other simulation training models, and virtual reality training could provide a useful training adjunct to integrate into surgical training curricula.

## Introduction

Laparoscopic cholecystectomy is the standard approach for gallbladder excision in patients with symptomatic gallstones^1,2^. The introduction of laparoscopic cholecystectomy in the 1980s provides an example of the challenges associated with adoption of new minimally invasive approaches and it initially resulted in an increase in bile duct injury internationally^3^. In this setting, new technologies such as simulation, can mitigate the learning curve of surgical trainees. Simulation training (ST) within surgical practice is any activity that aims to imitate an environment to inform, modify or assess skills and behaviours^4,5^. The creation of the environment can be either physical or virtual. Physical environments include dry- and wet-laboratory models, cadaveric (commonly porcine/human) or live, anaesthetized porcine, whereas virtual environments are computer-generated and viewed digitally^6^. In the Netherlands, the introduction of laparoscopic cholecystectomy initially resulted in a reduction of caseload for trainees but was then formally integrated into the training programme within 2 years^7^. Since then, there has been significant development of simulation to augment training in laparoscopic cholecystectomy, including porcine^8^, cadaveric^9^ and high-fidelity model simulation^10^. However, variability in access and quality can still be a barrier to ST^11^. Virtual reality training (VRT) is a potential solution that provides trainees with an opportunity to practice cognitive and technical skills outside of the operating theatre. Systematic reviews and meta-analyses have found that that operating time was significantly shorter and performance was improved in the virtual reality (VR) simulation groups compared with no supplementary training for surgical trainees in cross-specialty laparoscopic surgery^12,13^.

Laparoscopic cholecystectomy is an index procedure that is mandatory in international general surgery curriculae^7,14–17^, and is a common procedural module in VR simulation systems. In the research setting, operating performance can be measured by global rating scores (GRSs) and error scores^18^ and has also been inferred by total operating time^19,20^. Two validated and frequently used scores are Objective Structured Assessment of Technical Skill (OSATS)^21^ and Global Operative Assessment of Laparoscopic Skills (GOALS)^22^. OSATS was originally validated for direct observation in open surgery, and later validated in laparoscopic cholecystectomy^18,21,23^. GOALS was developed and validated to assess the specific skilled required for laparoscopic surgery, including direct and delayed observation. GOALS direct observation includes the domain autonomy^18,22,23^. In an RCT comparing OSATS and GOALS scores as assessment tools for laparoscopic cholecystectomy, mean OSATS and GOALS score were found to have high correlation^23^.

This manuscript sought to perform a meta-analysis of trials evaluating VRT *versus* simulated training (ST) or no additional training (NAT) for laparoscopic cholecystectomy.

## Methods

### Design and search strategy

A systematic review and meta-analysis were performed in concordance with the PRISMA guidelines^24–26^ and with reference to the Cochrane Handbook^27^. The study was registered prospectively on PROSPERO (ID CRD42020208499).

The following PICO was used:

Population: junior trainees (medical students, core trainees, foundation trainees, senior house officers, registrars, residents and general surgery trainees).

Intervention: VRT (basic skills and laparoscopic cholecystectomy procedural skills).

Comparator: NAT or ST.

Outcome: performance measured by a GRS (OSATS and GOALS) and time to completion of task.

A systematic literature search was undertaken on the following databases: MEDLINE (PubMed), Embase (Ovid SP), Web of Science, Scopus and LILACS using the following medical subject headings and free-text keywords in combination: ‘laparoscopic cholecystectomy, ‘virtual reality’ and ‘laparoscopic surgery’. The complete search string is available in *Table S1*. All abstracts, studies and citations identified were reviewed for suitability, initially by title and abstract, and subsequently by full text where appropriate and an inclusion and exclusion criteria was applied. The reference lists of eligible studies were searched further to identify any additional relevant studies. All languages were considered, with no restrictions placed on date of publication or publication status. The first search was performed on 4 August 2020 and repeated on 25 November 2020. E-mail alerts were created for searches to identify further publications. A final search was conducted before submission on 7 January 2022. Randomized clinical trials (RCTs) comparing VRT with either NAT or ST or operating theatre training for surgical trainees and medical students were included. Studies were only included if the performance outcome was measured in a laparoscopic cholecystectomy. Studies were included if the VR training was either basic tasks, procedural tasks, or both. Studies that assessed performance outcomes on basic skills and non-randomized validation trials were excluded. A full list of inclusion and exclusion criteria is available in *Table S2*.

### Quality assessment

The Cochrane Risk of Bias Tool^28^ (the Nordic Cochrane Centre, The Cochrane Collaboration, Copenhagen, Denmark) was applied to determine the quality of each eligibility study objectively. Where risk of methodological bias was not clearly explained, risk was considered ‘high’ for the purpose of reporting.

### Definitions and data categorization

Data were pooled for analysis over the following defined categories:

#### Junior Trainees

General surgery trainees (senior house officers, registrars and residents), foundation trainees (house officers and interns), and medical students were analysed together. The limited early experience of some individuals was assumed to have a low impact on results and reflects the varying abilities of a cohort of junior trainees.

#### Virtual reality training

VRT refers to computer-generated environments to rehearse surgical skills and it must be noted that studies used several VRT systems. Studies mostly used basic skills training such as hoops on pegs and laparoscopic cholecystectomy procedural training modules. The difference between models and versions was assumed with a low impact on results.

#### No Additional Training

Studies compared VRT with NAT. It is possible that general surgery trainees (residents) while not receiving additional training as part of the study, would also be receiving their standard training in the operating theatre concomitantly. Although, the impact of this variability is unknown, it was assumed as a reflection in the variability in training and trainees between centres.

#### Simulation Training

This refers to non-computer-generated environments to rehearse surgical skills. Studies comparing ST describe a widely available variety of laparoscopic box trainers (BTs) with different designs and materials and accompanying didactic teaching and e-learning resources, reflecting the variability in ST available between centres.

#### Laparoscopic Cholecystectomy

Some studies assessed participants at multiple time points following intervention. In this case, the first assessment only point was used in meta-analysis. All initial assessment laparoscopic cholecystectomies were analysed together, including human and live, *in vivo* and *ex vivo* porcine models. Participants were assessed at different post-intervention times between studies. Within each study the post-intervention assessment time was consistent within and between groups.

### Outcomes of interest

Operative performance GRS collected during the first post-intervention laparoscopic cholecystectomy was the primary outcome of interest. Other quantifiable performance metrics (such as time to completion of task) were included. With respect of the time to completion of task, not all the studies measured the task start and finish time; some studies measured the start/end of intraoperative phases. The term ‘time to completion’ was used to describe the total time of assessment.

For meta-analysis, total OSATS, total GOALS, individual domains of GOALS for delayed observation and time to completion of task were used. *Tables S3 and S4* detail the domains, descriptors and scoring for OSATS and GOALS respectively.

### Data extraction

Two reviewers independently performed the literature search and eligibility assessment. The same two reviewers independently extracted the data from the included studies. Extracted data included first named author, year of publication, country the study was conducted, study design, inclusion and exclusion criteria, GRS used and time to completion of task. Additional details were recorded, including the demographics and training history of the participating trainees, the VR system used, the basic and procedural task performed for training and the method and study time for performance assessment. Mean(SD) values for continuous data were extracted in the post-intervention study. Where numerical values were not provided data were extracted from figures using WebPlotDigitizer version 4.4^29^. Any discrepancies in eligibility or data extraction were resolved by consensus and with a third author. Where necessary, mean(SD) were estimated from the available median, interquartile range (IQR) and confidence interval (CI) or range using standard approaches^30^. In summary, the median value was considered as the mean(SD) calculated as IQWR/1.35; (95%CI)/3.92; range/4^30,31^.

### Statistical analysis

An *a priori* plan to meta-analyse GRS and time to completion of task between those who received VRT compared with NAT and VRT compared with ST was made. GRS suitable for inclusion were reported OSATS or GOALS scores, which use continuous scales (minimum 1 and maximum 5), per category of assessment and reported time to completion of task in minutes. These outcomes were treated as continuous data. Review Manager version 5.3 (the Nordic Cochrane Centre, The Cochrane Collaboration, Copenhagen, Denmark) was used to perform the meta-analysis and to generate forest and funnel plots. Pooled data analysis was reported as mean difference (MD) values and 95 per cent confidence intervals were calculated. The MD was the difference in GRS or difference in time to completion of task between groups. The *I*^2^ statistic was used to examine the heterogeneity among effect estimates in included studies. Significant statistic heterogeneity among studies was defined as an *I*^2^ statistic greater than 50 per cent^32^. A fixed-effects model (using Mantel–Haenszel methods) was used when there was significant statistical heterogeneity, and a random-effects model (using inverse variance methods) was used when there was no significant heterogeneity.

Publication bias was estimated visually through generation of funnel plots for outcomes that were significant following pooled analysis. Funnel plots were generated as a function of sample size against effect size. Each point on the graph represents a standardized comparison of an individual study comparing the outcome effect with the MD with the SE of the MD [SE(MD)].

## Results

**Fig. 1:**
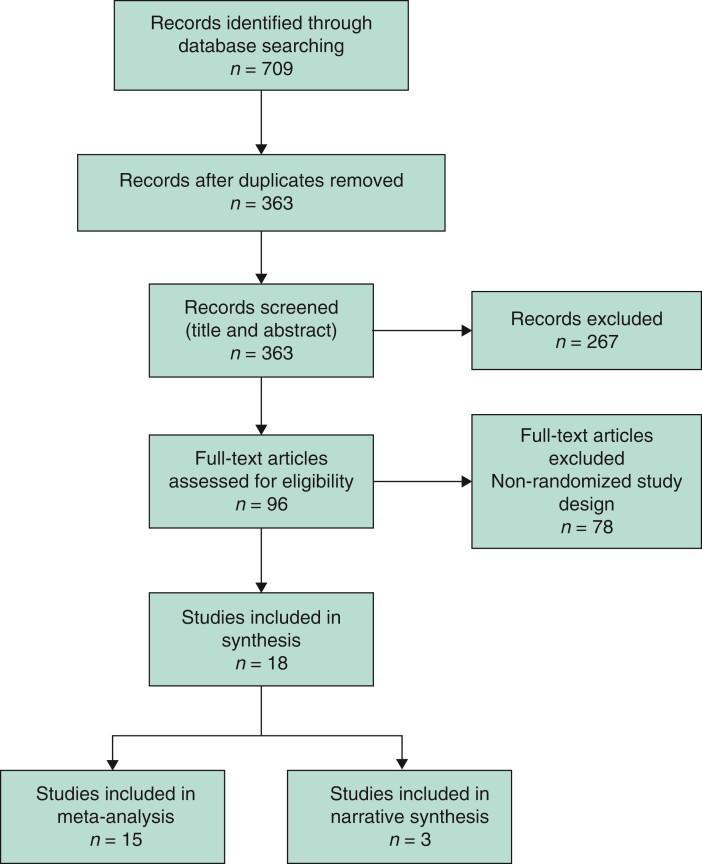
PRISMA diagram

The results of the literature search are shown in *Fig. 1*. After exclusion of duplicates, 363 references were screened by title and abstract. Ninety-six full-text articles were reviewed, and 18 RCTs that met the inclusion criteria for synthesis were identified. Fifteen studies^8,33–42^ were included for meta-analysis and 3 further studies^43–45^ for narrative synthesis only.

**Fig. 2:**
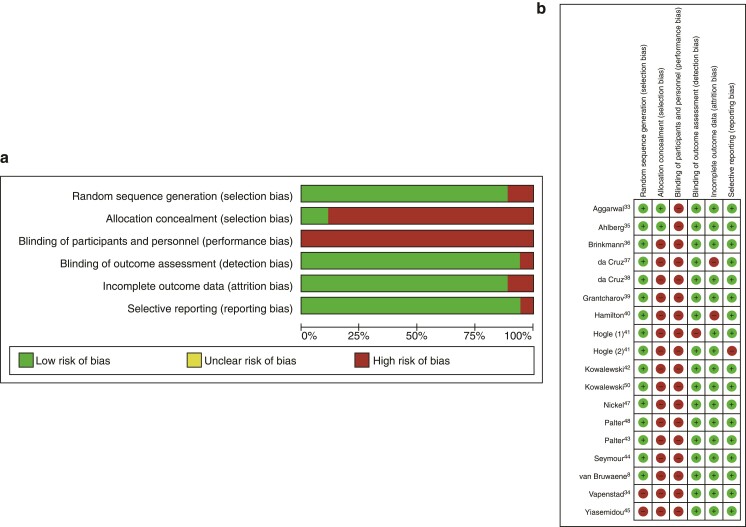
Risk of bias

Characteristics of the included studies are summarized in *Table 1* and *Table S5*. Thirteen studies were prospective, single-centre, two-arm RCT^33,34,37,38,40,41,43–49^, 2 were single-centre, three-arm RCT^8,50^ and 3 were multicentre, two-arm RCT^35,39,41^. Thirteen studies compared VR training and NAT. Of these, six studies reported on GRSs and times^34,37–39,46,50^, three studies reported on GRS only^41,43^, two studies reported on GRS and VR metrics and times^33,48^, one study reported error score only^35^ and one study reported error scores and time^44^. Four studies compared VR training and ST. Of these, two studies reported GRS only^40,49^, one study reported GRS and times^47^ and one study reported GRS and VR metrics^45^. One study compared VR training with NAT and ST and reported GRS only^8^. Risk of bias within individual studies ranged from low to moderate (*Fig. 2*) with high risk of performance bias due to the non-blinding of participants to the intervention, followed by selection bias due to non-blinding of members of the study team to the intervention allocation of the participants. There was a low risk of detection bias as most studies used delayed video assessment of more than one assessor blinded to the intervention. Risk of publication bias was assessed using funnel plots and significant results are presented with the corresponding forest plot in *Figs. S1* and *S2*, documenting that the risk of publication bias is low.

**a** Types of bias present. **b** Bias by study.

**Table 1:** Summary of included studies

First author (year), country	Study design	Participant grade (total)	Intervention(s):control	VR system and tasks	Comparator	Assessment	Outcomes	Assessment of IRR	Result: which training was favoured?
**Aggarwal (2007)^33^ UK**	SC, 2-RCT	Residents* (20)	10:10	LapSim Basic and procedural	NAT	First 5 porcine cadaveric LCs post-intervention	OSATS, time, video motion analysis system	Cronbach’s α 0.74	VRT
**Ahlberg (2007)^35^ Sweden**	MC, 2-RCT	Residents* (13)	U:U	LapSim 2.0 Basic and procedural	NAT	First 10 LCs in the OT post-intervention	Seymore error score	κ 0.8	VRT
**Brinkmann (2017)^49^ Germany**	SC, 2-RCT	Medical students (36)	18:18	Lap Mentor II Basic and procedural within a 5-day curriculum	ST basic task within 5-day curriculum	Post-intervention, *ex vivo* porcine LC model	GOALS	Cronbach’s α 0.855 ICC >0.75	ST
**da Cruz (2010)^37^ Brazil**	SC, 2-RCT	Medical students (15)	5:5:5 (UC)	LapVR Basic and procedural	NAT	Post-intervention, *in vivo* porcine LC (UClive or cadaveric)	GOALS, procedure score, time, and blood loss	U	NSD
**da Cruz (2016)^38^ Brazil**	SC, 2-RCT	Medical students (20)	10:10	LapVR Basic	NAT	Post-intervention, *in vivo* live anaesthetized porcine LC	GOALS, intraoperative phase time and blood loss	U	VRT
**Grantcharov (2004)^39^ Canada**	MC, 2-RCT	Residents† (20)	10 enrolled (8 analysed) : 10 enrolled (8 analysed)	MIST VR Basic	NAT	Pre- and post-intervention LC in the OT	Other GRS, time	Cohen’s κ 0.71	VRT
**Hamilton (2002)^40^ USA**	SC, 2-RCT	Residents‡ (50)	25:25	MIST VRBasic	ST	Pre- and post-intervention LC in the OT	Other GRS	U	VRT
**Hogle Study 1 (2009)^41^ USA**	MC, 2-RCT	Residents§ (13) Attrition 1-UC (which group randomized to)	6:6	LapSim Basic	NAT	First 2 LCs in the OT post-intervention	GOALS, time	U	NSD
**Hogle Study 2 (2009)^41^ USA**	SC, 2-RCT	General surgery interns (21)	10:11	LapSim Basic	No training	Post-intervention, *ex vivo* porcine LC model	GOALS, time	U	VRT
**Kowalewski (2018)^42^ Germany**	SC, 2-RCT	Residents* (64)	33 8 analysed: 31	Lap Mentor II Basic and procedural (with BT and 3D training)	Standard resident training	Pre- and post- *ex vivo* porcine LC model on POP trainer	GOALS, time	Not performed	VRT
**Kowalewski (2019)^50^ Germany**	SC, 3-RCT	Medical students (100)	Alone 40: Dyad 40:20	Lap Mentor II Basic and procedural (with online training)	Alone *versus* Dyad *versus* no training	Post-intervention *ex vivo* porcine LC model on POP trainer and VR LC	OSATS, GOALS, time	Not performed	VRT
**Nickel (2015)^47^ Germany**	SC, 2-RCT No control arm	Medical students (84)	42:42	Lap Mentor II Basic and procedural (with online training)	ST	Post-intervention *in vivo* cadaveric porcine LC	OSATS, time	U	Mixed
**Palter (2014)^43^ Canada**	SC, 2-RCT	Residents† (16)	8:8 (UC)	LapSim 2.0 Basic and procedural	NAT	Pre- and post-intervention LC in the OT	OSATS	U	VRT
**Palter (2013)^48^ Canada**	SC, 2RCT	Residents* (20)	10:8 (UC)	LapSim Basic and procedural (with cognitive training, BT, and OT participation)	NAT	First 5 LCs in the OT post-intervention and VR LC	OSATS, LapSim metrics	U	VRT
**Seymour¶ (2002)^44^ USA**	SC, 2-RCT	Residents§ (18)	8:8 (UC)	MIST VR	NAT	Single LC in the OT post-intervention	Error score	Percentage agreement 91(4)%	VRT
**Van Bruwaene (2015)^8^ Belgium**	SC, 3-RCT	Medical students (30)	10:10:10	Lap Mentor Basic and procedural	NAT/ST	Post-intervention live anaesthetized porcine LC (1 week, 4 months)	GOALS, time	1/3 raters significantly different	NSD
**Våpenstad (2017)^34^ Norway**	SC, 2-RCT	Medical students and interns (30)	16:14	LapSim with Xitact IHP haptics Basic	NAT	Post-intervention, *ex vivo* porcine LC model	GOALS	Significantly different between 2 raters in bimanual dexterity	NAT
**Yiasemidou (2017)^45^ UK**	SC, 2-RCT	CT1-ST5 (25) Attrition? group	7:9	Lap Mentor Basic	Box trainer	Pre- and post- *ex vivo* porcine LC model and VR LC	GOALS Lap Mentor metrics	ICC = 0.894 95% c.i. 0.849–0.925	Mixed

* Laparoscopically inexperienced as primary surgeon. †Limited experience as primary laparoscopic surgeon. ‡Year 1–2. §Year 1. ¶PGY1–4. 2-RCT, two-arm randomized control trial; 3-RCT, three-arm randomized control trial; BT, box trainer; GOALS, Global Operative Assessment of Laparoscopic Skills; ICC, intraclass coefficient; LC, laparoscopic cholecystectomy; MC, multicentre; NAT, no additional training; NSD, no significant difference; OSATS, Objective Structured Assessment of Technical Skills; OT, operating theatre; POP, pulsatile organ perfusion trainer; SC, single centre; ST, simulation training; U, unknown; UC, uncertain; VT, video trainer; VR, virtual reality; PGY, post-graduate year; CT1-ST5, core- medical trainee 1st year - specialty trainee 5th year. Greyed rows indicate studies not included in meta-analysis.

### Virtual reality training *versus* no additional training

**Fig. 3:**
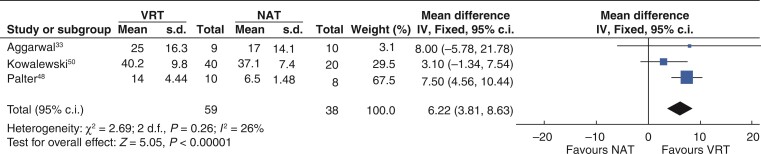
Forest plot for metanalysis total Objective Structured Assessment of Technical Skills (OSATS) scores from randomized clinical trials comparing VR training (VRT) and no additional training (NAT)

Four studies reported OSATS scores in RCTs comparing VRT and NAT^33,43,48,50^. In a meta-analysis of three studies^33,48,50^, 59 participants were randomized to VRT and 38 to NAT. Statistical heterogeneity was low (*I*^2^ = 23 per cent). Using a fixed-effects model, the combined weighted effected favoured VR training over NAT (MD = 6.22, 95 per cent c.i 3.81 to 8.36, *P* < 0.00001) as shown in *Fig. 3*. A single-centre RCT^43^ randomized 16 participants (general surgery trainees and residents) to conventional residency training or deliberate practice on a VR simulator. The curricula tasks in the deliberate practice group were prescribed by individual feedback. The participants were assessed performing a laparoscopic cholecystectomy in the operating theatre. Significantly higher OSATS score were found in the deliberate practice group compared with the control group (median 17.0, i.q.r. 15.3–18.5 *versus* median 12.5, i.q.r. 7.5–14.0, *P* = 0.03)^43^. This study was excluded from meta-analysis because the VR training received was personalized following assessment and feedback, which was not comparable to other interventions.

Eight studies compared reported GOALS score in RCTs comparing VRT and NAT^8,34,37,38,41,42,50^. Three studies reported total GOALS^8,46,50^. Five studies reported the GOALS domains: depth perception bimanual dexterity, efficiency and tissue handling^34,37,38,41^.

**Fig. 4:**
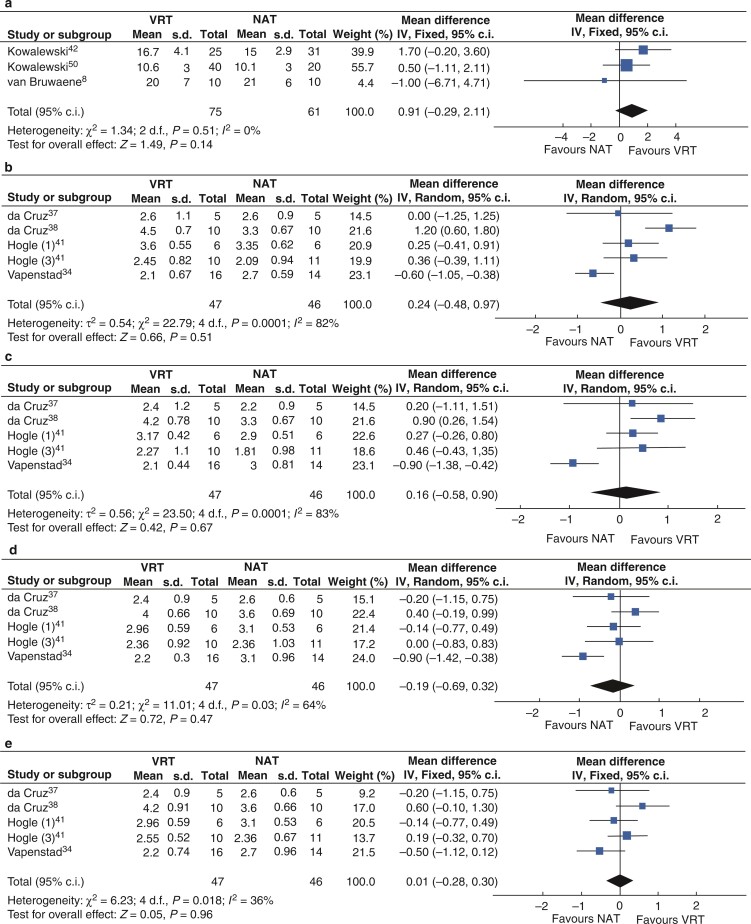
Forest plot for metanalysis of Global Operative Assessment of Laparoscopic Skills (GOALS) scores from randomized clinical trials comparing VR training (VRT) and no additional training (NAT)

Total GOALS scores were analysed including in three studies^8,46,50^, where 75 participants were randomized to VRT and 38 participants to NAT. Statistical heterogeneity was low (*I*^2^ = 0 per cent) using a fixed-effects model, the combined weighted effected favoured neither VRT nor NAT (MD 0.91, 95 per cent c.i. −0.29 to 2.11, *P* = 0.14) as shown in *Fig. 4*.

**a** Total. **b** Depth perception. **c** Bimanual dexterity. **d** Efficiency. **e** Tissue handling.

In a meta-analysis of five studies^34,37,38,41^, where 47 participants were randomized to VRT and 46 to NAT, GOALS domain scores were analysed.

For the GOALS domain of depth perception, statistical heterogeneity was substantial (*I*^2^ = 82%). Using a random-effects model, the combined weighted effect favoured neither VRT nor NAT (MD = 0.24, 95%CI.−0.48 to 0.97, *P* = 0.51). For bimanual dexterity, statistical heterogeneity was moderate (*I*^2^ = 83%). Using a random-effects model, the combined weighted effect favoured neither VRT nor NAT ([MD = 0.16, 95% 0.58 to 0.90, *P* = 0.67). With respect of the GOALS domain efficiency, statistical heterogeneity was substantial (*I*^2^ = 64%). Using a random-effects model, the combined weighted effect a favoured neither VRT nor NAT (MD −0.69, 95&CI. −0.69 to 0.49, *P* = 0.47). Finally, for the GOALS domain of tissue handling, statistical heterogeneity was moderate (*I*^2^ = 36%). Using a fixed-effects model, the combined weighted effect a favoured neither VRT nor NAT (MD = 0.01, 95%CI −0.28 to 0.52, *P* = 0.30) as shown in *Fig. 4*.

Two studies reported GRSs other than OSATS and GOALS^39,40^. A single-centre RCT^40^ randomized 50 participants to either VRT or ST using a video (box) trainer. Performance was assessed on either a VR simulated laparoscopic cholecystectomy or a laparoscopic cholecystectomy in the operating theatre (*n* = 19). This study used an author’s GRS, who later published OSATS. This study found no statistically significant difference in pre- and post-intervention performance in either group, nor between the VR training and simulation groups^40^. Another single-centre RCT^39^ randomized 20 participants to either VR training or NAT. Participant’s performance was reported using the authors’ GRS. Which, like OSATS and GOALS features five domains (economy of movement—unnecessary movements, economy of movement—confidence of movements, errors—respect for tissue, errors—precision of technique), for each of which the participant can score 1–5. Eight participants in each arm of the trial were analysed. This study compares each group’s GRS when performing a laparoscopic cholecystectomy in the operating theatre pre- and post-intervention. This study found that the VR group had significantly greater improvement in their economy of movement (*P* = 0.003) and error (*P* = 0.003) GRSs compared with the control group^39^.

**Fig. 5:**
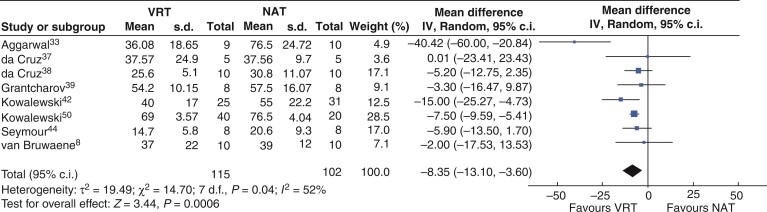
Forest plot for metanalysis time to completion of task (minutes) from randomized clinical trials comparing VR training (VRT) and no additional training (NAT)

In a meta-analysis of eight studies^8,33,37–39,42,44,50^, 115 participants were randomized to VR training and 102 to NAT. Statistical heterogeneity was substantial (*I*^2^ = 52%). Using a random-effects model, the combined weighted effect favoured VRT (MD=−8.35 min, 95 per cent c.i. 13.10 to 3.60, *P* = <0.001) as shown in *Fig. 5*.

Two studies reported surgical error^35,44^. The error reporting system, used by both studies, details eight error definitions (lack of progress, gallbladder injury, liver injury, incorrect plane of dissection, burn to non-target tissue, tearing tissues, instrument out of view, and attending takeover)^44,51^. A single-centre two-arm RCT^44^ randomized 16 participants to either VR training or NAT and assessed participants performance a laparoscopic cholecystectomy on an *ex vivo* porcine model in a BT. There were statistically significant differences in mean error counts between the VRT and NAT groups (1.19 *versus* 7.38, *Z* = −2.76, *P* < 0.006 Mann–Whitney *U* test). This study also reported that gallbladder injury and burns to non-target tissues were five times more likely to occur in the no additional training group than the VRT group and participants in the NAT group were nine times more likely to be scored as a lack of progress. This study reported one liver injury (VRT group) and no tearing errors. Excluding the liver injury, the NAT group made significantly more total errors (six times more than the VRT group)^44^. This study additionally reported that the VRT group completed the assessment laparoscopic cholecystectomy faster than the NAT group (29 per cent faster), these data was included in meta-analysis (*Fig. 5*). Another single-centre two-arm RCT^35^ randomized 13 participants to either VRT or NAT and assessed the first 10 laparoscopic cholecystectomy cases in the operating theatre. Thirty-seven procedures were assessed in total. Significant differences were found in mean error counts between the VRT group, who made fewer intraoperative errors, compared with the NAT group (mean 28.4, 95 per cent c.i. 23.51 to 33.32 *versus* mean 86.2, 95 per cent c.i. 58.18 to 114.12, *P* = 0.0037)^35^. It was not possible to successfully extract first laparoscopic cholecystectomy error counts from the published figures. No significant difference was found between groups in the analysis of intraoperative phases: exposure, clipping and tissue division and dissection. Meta-analysis of error scores from these two studies was considered; however, the pooled analysis of errors counted in a complete human laparoscopic cholecystectomy and part of an *ex vivo* porcine model was deemed not appropriate considering the sizeable difference in results and operative times.

### Virtual reality training *versus* simulation training

**Fig. 6:**
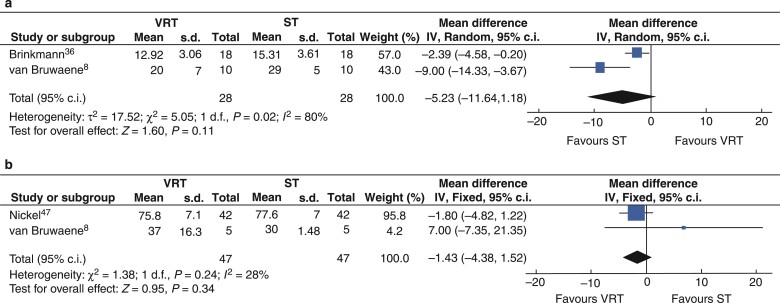
Forest plot for metanalysis of scores from randomized clinical trials comparing VR training (VRT) and simulation training (ST)

In a meta-analysis of two studies^8,36^, 28 participants were randomized to VRT and 28 to ST. For total GOALS score, statistical heterogeneity was considerable (*I*^2^ = 80 per cent). Using a random-effects model, the combined weighted effect favoured neither VRT nor ST (MD −5.23, 95 per cent c.i. −11.64 to 1.18, *P* = 0.11) as shown in *Fig. 6*.

**a** Total Global Operative Assessment of Laparoscopic Skills (GOALS). **b** Time to completion of task (minutes) from RCT comparing VRT and ST.

A single-centre, two-arm randomized intervention trial^47^ randomized 84 participants to either VRT or ‘blended learning’ (structured ST and e-learning) as assessed participants on six VR laparoscopic cholecystectomy cases with different clinical histories and anatomical variations and a cadaveric porcine model (‘pulsatile organ perfusion (POP) trainer’). Nineteen participants in the VRT group completed a cadaveric porcine model laparoscopic cholecystectomy, compared with 9 in the blended learning/simulation group. No significant difference was found in total OSATS scores (measured on the cadaveric model) between intervention groups. (49.5(10.5) *versus* 49.7(12.0), MD 0.3, 95 per cent c.i. −4.7 to 5.3)^47^. These data could not be included in the meta-analysis as there were no similar studies reporting OSATS. A further single-centre, two-arm randomized intervention trial^45^ randomized 25 participants to either VRT or ST (a BT that could be used at home). Sixteen participants completed the study (VRT = 7, ST = 9), who were assessed on both a VR laparoscopic cholecystectomy and a BT simulation model. GOALS scores were reported for the laparoscopic cholecystectomy BT assessment. This study reports a statistically significant improvement in median total GOALS in the VRT group compared with the ST group; however, this was not seen in the individual GOALS domains scores^45^. This study was not included in meta-analysis as there were insufficient data reported to calculate the mean and s.d.

In a meta-analysis of two studies^8,47^, 47 participants were randomized to VR training and 47 to ST. Statistical heterogeneity was low (*I*^2^ = 28 per cent). Using a fixed-effects model, the combined weighted effect favoured neither VRT nor ST (MD −1.43, 95 per cent c.i. −4.43 to 1.52, *P* = 0.34) as shown in *Fig. 6*.

## Discussion

This systematic review and meta-analysis suggests that VRT improves OSATS scores and decreases time to completion of task in laparoscopic cholecystectomy compared with NAT for junior trainees. No significant increase in GOALS scores was found.

Improved OSATS score was seen in several studies. In one study, comprehensive training accompanied the VRT, which could account for the differences seen. One study demonstrated a significantly higher mean OSATS in their intervention group, which consisted of case-based learning, proficiency-based VRT, laparoscopic box training, and operating theatre participation^48^. In another study, the intervention group received VRT, online technical and procedural skills modules, and an interval of familiarization with VR and BT; however, the higher OSATS score in this intervention group in did not reach significance^50^. In contrast, a different study’s intervention was limited to basic and procedural VRT, with no accompanying didactic teaching. The reported results suggest a greater variation of OSATS scores. It could be the training accompanying the VRT was responsible for the improvement in performance. Considering total GOALS score, two of the three included studies VRT interventions were accompanied by didactic teaching^42,50^. In one study the intervention group received didactic teaching on only the procedural VRT. The results reported variable mean increases in total GOALS score^8^. There was variation in the three expert raters contributing to this study^8^, which may have affected mean scores used for meta-analysis. A further study demonstrated significant improvement in economy of movement and efficiency scores in the VRT group compared with the NAT group^39^. Participants in the VRT group received basic skills training and just part of the procedure was assessed. In not assessing the dissection of the hepatocystic triangle, the authors have excluded a crucial intraoperative phase that has been found to have a higher error rate for both trainees and consultants (attendings)^52,53^.

Despite the improvement in OSATS there was no improvement in GOALS. A lack of a significant difference in tissue handling scores could represent a lack of haptic feedback. In robotic surgery it is possible to substitute visual feedback for haptic feedback, but only in experienced surgeons^54^. One study included haptic feedback in VR simulation^34^ and found that the NAT group achieved significantly higher GOALS scores across the domains of depth perception, bimanual dexterity, and efficiency^34^; however, it has been suggested that simulated haptic feedback can hinder an inexperienced surgeon learning a new task^55^.

No significant difference in total GOALS and time to completion of task in laparoscopic cholecystectomy in junior trainees comparing VRT and ST was found. It is possible that the number of studies and sample sizes were not large enough to demonstrate the difference.

Outcomes were assessed in both simulated environments and in the operating theatre, which could have influenced the performance of the participants. Six studies comparing VRT and NAT assessed participants in the operating theatre with human laparoscopic cases^35,39,41,43,44,48^. Of these studies, only one found no significant difference between groups^41^, with all other studies favouring VRT^35,39,44,48^. The studies that compared VRT and NAT by assessing participants on *ex vivo* porcine models^34,41,42,50^ also favoured VRT^41,42,50^ over NAT^34^.

An advantage of VRT is self-directed training, although training in pairs has been shown to be more efficacious than training alone^50^. VRT can be delivered without direct trainer supervision, utilizing the VR systems feedback and metrics, providing opportunities for trainees to take greater ownership of aspects of their training. There are fewer safety and staffing implications with VRT. The cost of VR systems is considered a disadvantaged of VR training and is often cited as barrier to implementation. There is a paucity of cost–benefit analysis evidence to support this or pertaining to surgical training generally^56,57^. Modern VR systems cost 4500–100 000 Euro^58^, and while this is a significant financial investment, it should be balanced against the benefits (and contributions) of cross-specialty use and potential reduction in junior operating times^20^ and in costs of morbidity^59^. This cost should also be compared with the overhead costs of housing, equipping, staffing, maintaining, and running a traditional simulation centre^60^; the cost of which rises when live animal/cadaveric simulation is delivered^60^. Many of these costs are often incurred by the trainees who are required to contribute to the cost of attending simulation courses^61^. Patients, as key stakeholders in surgical training and curriculum development, take interest and consider surgical ST crucial^62,63^.

This study has a few limitations. It was not possible to include all studies in meta-analysis, and a few studies with small sample sized and methodological heterogeneity necessitated pooled analysis. While this synthesis may limit the conclusions that can be drawn and can increase the risk of increase the risk of type II errors, it could consider a pragmatic reflection of the heterogeneity or inequality in training and access to training materials faced by trainees. Some studies did not report numerical values in their results. Although data were extracted from figures using software, values were checked independently, and mean and s.d. values were calculated using a published methodology, it is possible that the extracted data may not match the original data. While this study shows some improvement in surgical performance in research settings, with diverse assessments, it is not possible to conclude direct translation to routine clinical practice. This limits the interpretation of results.

VRT in laparoscopic cholecystectomy is in an important area for ongoing research. This could include comparing different virtual reality models, including basic and procedural skills, to confer improved skill acquisition in laparoscopic surgery. Large multicentre trials would be useful to examine the potential benefit of VRT to surgical training, or smaller trials could consider their methodology and study power to facilitate future meta-analysis.

It is difficult to remove selection and performance bias in these studies as interventions cannot be concealed from participants. While the allocation of participants is not always concealed to researchers, analysing quantitative data generated by video reviewers to whom the participant allocations are concealed, reduces detection bias.

VRT may improve performance and reduce operating time compared with NAT in laparoscopic cholecystectomy for junior surgical trainees and could provide a training adjunct for surgical training curricula.

## Supplementary Material

zrac086_Supplementary_Data

## Data Availability

The data analysed in this study are available in the published articles cited. Data generated by this analysis are available in the article and supplementary material and data extracted from figures and calculations can be shared on reasonable request to the corresponding author.
