# Supplementary material for: The impact of virtual reality simulation training on operative performance in laparoscopic cholecystectomy: meta-analysis of randomized clinical trials
Source: BJS Open. 2022 Jul 18;6(4):zrac086. doi: 10.1093/bjsopen/zrac086 (PMC9291386; doi:10.1093/bjsopen/zrac086)
Supplement: zrac086_Supplementary_Data [file zrac086_supplementary_data.zip › Supplementary_material.docx]

## **Supplementary material**

## **Table S1. Systematic literature search string**

| Search terms |
| --- |
| Laparoscopic cholecystectomy (MeSH)* |
| Virtual reality (MeSH)* |
| Laparoscopic cholecystectomy (keyword) |
| Virtual reality (keyword) |
| 1* OR 3 |
| 2* OR 4 |
| 5 AND 6 |
| *To be performed in databases which allow MeSH term searches |

**Table S2. Systematic review inclusion and exclusion criteria.**

|  | Inclusion Criteria | Exclusion Criteria |
| --- | --- | --- |
| Study design | Interventional studies  Randomised  Laparoscopic cholecystectomy | Reviews  Other laparoscopic surgery  Non-randomised |
| Population | Medical students  Surgical trainees (any stage)  Surgical residents (any stage) | Consultants |
| Intervention | Virtual reality simulation |  |
| Comparison | Simulation (any) training including technical and cognitive skills  Supervised clinical training in the operating theatre | Online tutorials/webinars  Cognitive simulation without virtual reality |

| Domain | Global rating scale  1 2 3 4 5 | | |
| --- | --- | --- | --- |
| Respect for tissue | Frequently used unnecessary force on tissue or caused damage by inappropriate use of instruments | Careful handling of tissue but occasionally caused inadvertent tissue damage | Consistently handled tissues appropriately with minimal damage |
| Time and motion | Many unnecessary moves | Efficient time/motion but some unnecessary moves | Economy of movement and maximum efficiency |
| Instrument handling | Repeatedly makes tentative awkward moves with instrument | Competent use of instruments although occasionally appeared stiff awkward | Fluid moves with instruments and no awkwardness |
| Knowledge of instruments | Frequently asked for the wrong instrument or used an inappropriate instrument | Knew the names of most instruments and used appropriate insert for the task | Obviously familiar with the instruments required and their names |
| Use of assistants | Simply placed assistants poorly or failed to use assistants | Good use of assistants most of the time | Strategically use assistant to the best advantage at all times |
| Flow of operation and forward planning | Frequently stopped operating or needed to discuss the next move | Demonstrated ability for forward planning with steady progression of operative procedure | Obviously planned course of operation with F at this flow from one move to the next |
| Knowledge of specific procedures | Deficient knowledge. Needed specific instruction at most operative steps | Knew all important aspects of the operation | Demonstrated familiarity with all aspects of the operation |
| Overall, on this task, should this candidate **PASS** or **FAIL**? | | | |

**Table S3. The Objective Structured Assessment of Technical Skills (OSATS)^19^**.

**Table S4. Global Operative Assessment of Laparoscopic Skills (GOALS)^20^**

|  | Global rating scale | | |
| --- | --- | --- | --- |
| Domain | 1 2 3 4 5 | | |
| Depth perception | Constantly overshooting target, hits backstop, wide swings, slow to  correct | ﻿Some overshooting  or missing plane, but corrects quickly | ﻿Accurately directs  instruments in correct plane to target |
| Bimanual dexterity | ﻿Use of 1 hand, ignoring nondominant hand, poor  coordination between hands | ﻿Use of both hands, but does not optimize  interactions between hands to facilitate  conduct of operation | ﻿Expertly uses both  hands in a complementary manner to provide optimal working exposure |
| Efficiency | ﻿Uncertain, much wasted effort, many tentative motions, constantly changing focus of  operation, or persisting at a task without  progress | ﻿Slow, but planned and  reasonably organized | ﻿Confident, efficient, and safe conduct of operation, maintaining focus on component of procedure until better done by another approach |
| Tissue handling | ﻿Rough, tears tissue by  excessive traction, injures adjacent structures, poor control of coagulation device (recoil), grasper frequently slips off | ﻿Handles tissues  reasonably well, with some minor trauma to  adjacent tissues (e.g., coagulation of liver,  causes unnecessary liver bleeding, occasional slipping of grasper) | Handles tissues very well with appropriate traction on tissues and negligible injury of adjacent  structures. Uses energy sources appropriately but not excessively |
| Autonomy | ﻿Unable to complete entire procedure, even in a  straightforward case and  with extensive verbal guidance | ﻿Able to complete  operation safely with moderate prompting | ﻿Able to complete operation independently without prompting |

**Table S5. Full details of included studies.**

| First author  (year)_ | Aim/objective (quote) | Study design | Country | Participants | VR system | VR tasks | Comparator | Assessment task | Metric | Results | Conclusion (Quote) |
| --- | --- | --- | --- | --- | --- | --- | --- | --- | --- | --- | --- |
| Aggarwal (2017) | To compare learning curves for laparoscopic cholecystectomy (LC) after training on a proficiency based virtual reality (VR) curriculum with that of a traditionally trained group. | Prospective, single-centre, single-blinded, two-arm RCT | UK | Residents (laparoscopically inexperienced as primary surgeon) | Lap Sim | Basic and procedural | No training | First 5 porcine cadaveric LCs post-intervention. | OSATS, time, video motion analysis system | Significantly shorter operating time in VR group, path length and number of movements and OSATS | ﻿A proficiency-based VR training curriculum shortens the learning curve on real laparoscopic procedures when compared with traditional training methods. This |
| Ahlberg (2007) | To investigate whether proficiency-based training on the LapSim VR system improves objectively assessed, intraoperative performance during the initial learning of LC, and if that improvement is persistent over time | Prospective, multicentre, single-blinded, two-arm RCT | Sweden | Residents (laparoscopically inexperienced as primary surgeon) | LapSim 2.0 | Basic and procedural | Standard resident training | First 10 LCs in the OT post-intervention. | Seymore error score | Significantly more errors in control group | ﻿The results of this study show that training on the VR simulator to a level of proficiency significantly improves intraoperative performance during a resident’s first 10 LCs. |
| Brinkmann (2017) | ﻿To find out which training method leads to better acquisition of basic skills as the first step of simulation training, before application of these skills in a simulated surgical procedure follows. | Prospective, single-centre, single-blinded, two-arm randomised intervention trial | Germany | Medical students | Lap Mentor II | Basic within 5-day curriculum | Box trainer basic task within 5-day curriculum | Post intervention, ex-vivo porcine LC model | GOALS | Significantly higher skills scores in BT group | ﻿Despite both systems having advantages and disadvantages, they can both be used for simulation training for laparoscopic skills. In the setting with 2 structured, validated and almost identical curricula, the box-trained group appears to be superior in the better transfer of basic skills into an experimental but structured surgical procedure. |
| da Cruz (2010) | ﻿To determine whether practicing surgical skills in a VRSS results in improved surgical performance. | Prospective, single-centre, single-blinded, three-arm RCT | Brazil | Medical students | LapVR | Basic and procedural | No training | Post intervention, in-vivo porcine LC (UC live or cadaveric) | GOALS, procedure score, time, and blood loss | No significant difference in technical performance. | ﻿VRSS training is assumed to be an effective tool for learning and practicing laparoscopic skills. In this study, we could not demonstrate that VRSS training resulted in improved surgical performance. It may be useful, however, in familiarizing surgeons with laparoscopic surgery. More effective methods of teaching laparoscopic skills should be evaluated to help in improving surgical performance. |
| da Cruz (2016) | To ﻿verify whether there is benefit in surgical performance with preoperatory warm-up using a VRSS. | Prospective, single-centre, single-blinded, two-arm RCT | Brazil | Medical students | LapVR | Basic | No training | Post intervention, in-vivo live anaesthetized porcine LC | GOALS, phase time and blood loss |  | ﻿The practice of preoperative warm-up training seems to benefit surgical performance even in subject with mild laparoscopic experience. ( |
| Grantcharov  (2004) | To investigate whether laparoscopic skills acquired in a virtual environment could be transferred to actual operations, and therefore to validate the role of VR simulation as a tool for surgical skills training | Prospective, multicentre, single-blinded, two-arm RCT | Canada | Residents (limited experience as primary laparoscopic surgeon) | MIST VR | Basic | Standard resident training | Pre- and post- intervention LC in the OT | Own rating scale | VR group showed significant reduction in operative time, error score and economy of movement scores. | ﻿Surgeons who received VR simulator training showed significantly greater improvement in performance in the OR than those in the control group. VR surgical simulation is therefore a valid tool for training of laparoscopic psychomotor skills and could be incorporated into surgical training programmes. |
| Hamilton (2002) | ﻿The purpose of our study was threefold. First, we evaluated whether a virtual reality trainer (MIST VR) helps to develop video, hand, and eye dexterity better than an intense laparoscopic VT skills curriculum. Second, we addressed the question whether skills are transferable between laparoscopic training systems. Third, we evaluated whether improvement in psychomotor skills translates into improved operative performance during laparoscopic cholecystectomy. Finally, we gathered subjective data regarding resident preference between the two training systems. | Prospective, single-centre, single-blinded, two-arm RCT | USA | Residents (2 | MIST VR | Basic | VT/Box trainer basic task | Pre- and post- intervention LC in the OT | Resnick | Significant improvement in skills score in VR group no significant differences between intervention and control post-test skills scores | ﻿Psychomotor skills improve after training on both VR and VT, and skills may be transferable. Furthermore, training on a minimally invasive |
| Hogel Study 1 (2009) | ﻿To determine the impact of computer simulation training on the clinical performance of laparoscopic cholecystectomy by junior surgical residents. | Prospective, multicentre, single-blinded, two-arm RCT | USA | Residents (first year) | LapSim | Basic | Standard resident training | First 2 LCs in the OT post-intervention. | GOALS | No significant difference in technical performance. | |
| Hogel Study 2 (2009) | ﻿To determine whether training on the LapSim simulator improved laparoscopic cholecystectomy operative performance in an animal model | Prospective, single-centre, single-blinded, two-arm RCT | USA | General surgery interns | LapSim | Basic | No training | Post intervention, ex-vivo porcine LC model | GOALS | Significantly higher depth perception score in intervention group | |
| Kowalewski (2018) | ﻿To evaluate benefits of a combined multi-modality training program for surgical residents | Prospective, single-centre, single-blinded, two-arm RCT | Germany | Residents (laparoscopically inexperienced as primary surgeon) | Lap Mentor II | Basic and procedural (with BT and 3D training) | Standard resident training | Pre- and post- ex-vivo porcine LC model on POP trainer | GOALS, time | Significant improvements in GOALS and time in intervention group. Significantly reduced time in intervention group compared to control. (No sig. difference in total GOALS) | ﻿Structured multi-modality training is beneficial for novices to improve basics and overcome the initial learning curve in laparoscopy as well as to decrease operation time for LCs in different stages of experience. |
| Kowalewski (2019) | ﻿To define whether laparoscopy training should be done alone or in pairs (known as dyad training) | Prospective, single-centre, single-blinded, three-arm RCT | Germany | Medical students | Lap Mentor II | Basic and procedural (with online training) | Alone vs Dyad vs no training | Post-intervention ex-vivo porcine LC model on POP trainer and VR LC | OSATS, GOALS time | Significantly shorter operative time in dyad vs control (porcine LC). Significantly shorter operative time in VR LC in alone and dyad vs control. Significantly fewer movements and reduced path length in alone/dyad vs control. | ﻿The curriculum provided trainees with the laparoscopic skills needed to perform LC safely, irrespective of the number of trainees per workplace. Dyad training reduced the operation time needed for LC. Therefore, dyad training seems to be a promising alternative, especially if training time is limited and resources must be used as efficiently as possible. Trial |
| Nickel (2015) | To compare VR training with low-cost BL for teaching the basic performance of laparoscopic cholecystectomy (LC) to laparoscopic novices in a standardized and structured training program within an adequately powered trial. | Prospective, single-centre, single-blinded, two-arm intervention trial | Germany | Medical students | Lap Mentor II | Basic and procedural (with online training) | E-learning + BT | Post -intervention in-vivo cadaveric porcine LC | OSATS, time | ﻿The VR group completed the LC significantly faster and more often within 80 min than BL The BL group scored higher than the VR group in the knowledge test Both groups showed equal operative performance of LC in the OSATS score | ﻿VR and BL can both be applied for training the basics of LC. Multimodality training programs should be developed that combine the advantages of both approaches |
| Palter (2014) | ﻿To investigate whether the individualized deliberate practice on a VR simulator results in improved technical performance in the OR. | Prospective, single-centre, single-blinded, two-arm RCT | Canada | Residents (limited experience as primary laparoscopic surgeon) | LapSim 2.0 | Basic and procedural | Standard resident training | Pre- and post- intervention LC in the OT | Modified OSATS | Significantly better technical performance in the intervention group | ﻿A curriculum of deliberate individualized practice on a VR simulator improves technical performance in the OR. This has implications to greatly improve the feasibility of implementing simulation-based curricula in residency training programs, rather than having them being limited to research protocols. |
| Palter (2013) | ﻿to assess the effect of participation in a structured comprehensive curriculum on technical performance and learning curves in the OR as compared with conventional residency training, and second, to investigate whether curricular participation can affect the development of nontechnical skills. | Prospective, single-centre, single-blinded, two-arm RCT | Canada | Residents (laparoscopically inexperienced as primary surgeon) | LapSim | Basic and procedural (with Cognitive training, BT and OTparticipation | Standard resident training | First 5 LCs in the OR post-intervention and VR LC | Modified OSATS and LapSim metrics | The STAC group outperformed the control group in the first, second, third and fourth LCs. The control group demonstrated a significant learning curve in the OR. | ﻿Participating in the STAC shifted the learning curve for a basic laparoscopic procedure from the operating room into the simulation laboratory. STAC-trained residents had superior technical proficiency in the OR and nontechnical skills compared with conventionally trained residents. |
| Seymore (2002) | To demonstrate that virtual reality training transfers technical skills to the operating room environment | Prospective, single-centre, single-blinded, two-arm RCT | USA | Residents PGY 1-4 | MIST VR | Basic | Standard resident training | Single LC in the OT post-intervention. | Seymore error score | Significantly less errors and reduced operative time in intervention group | ﻿The use of VR surgical simulation to reach specific target criteria significantly improved the OR performance of residents during laparoscopic cholecystectomy. |
| Van Bruwaene (2015) | ﻿compared the transfer of training for the laparoscopic cholecystectomy from porcine cadaver organs vs virtual simulation to surgery in a live animal model in a prospective randomized trial. | Prospective, single-centre, single-blinded, three-arm RCT | Belgium | Medical students | Lap Mentor | Basic and procedural | cadaveric/no training | Post-intervention live anesthetized porcine LC (1 week, 4 months) | TOTAL GOALS ONLY AND time | ﻿The virtual trainer group did not outperform the control group at any time. | ﻿For trainees who are proficient in basic laparoscopic skills, the long-term advantage of additional procedural training, especially on a virtual but also on the conventional organ training model, remains to be proven. |
| Vapenstad (2017) | To ﻿examine predictive validity of the LapSimVR simulator equipped with the Xitact IHP handles with haptic feedback | Prospective, single-centre, single-blinded, two-arm RCT | Norway | Medical students and interns | LapSim with Xitact IHP haptics | Basic | No training | Post intervention, ex-vivo porcine LC model | GOALS | Significantly higher skills scores in control group | ﻿The criterion-based training program did not transfer skills to the clinical setting. Poor mechanical performance of the simulated haptic feedback is believed to have resulted in a negative training effect |
| Yiasmidou (2017) | To ﻿compares the efficacy of unsupervised training (other than induction) on VRSS located in clinical skills centres and “take-home” BTs on the subsequent performance of cholecystectomy | Prospective, single-centre, single-blinded, two-arm intervention trial | UK | CT1-ST5 | Lap Mentor | Basic | Box trainer | Pre- and post- ex-vivo porcine LC model and VR LC | GOAL and lap mentor metrics | Significant improvement in GOALS efficiency score in BT group. Significant improvement GOALS overall score in VR group. Significant improvement in all VR simulator metrics in BT group and in TT in VR group. BT group practiced significantly more than VR group. | “take-home” BTs are a potential alternative to VRSS. The former is an attractive option for surgical training as they are more portable and cost-effective and can therefore be provided to each trainee at the beginning of their training with reduced financial burden on their local hospital. |

RCT Randomised control trial, BT Box trainer, GOALS Global Operative Assessment of Laparoscopic Skills, ICC intraclass coefficient, LC Laparoscopic cholecystectomy, MC Multi-centre, NAT no additional training, NSD no significant difference, OSATS Objective Structured Assessment of Technical Skills, OT Operating Theatre, POP pulsatile organ perfusion trainer, SC Single-centre, ST simulation training, U unknown, UC uncertain, VT Video trainer, VR Virtual reality

Supplementary Figures


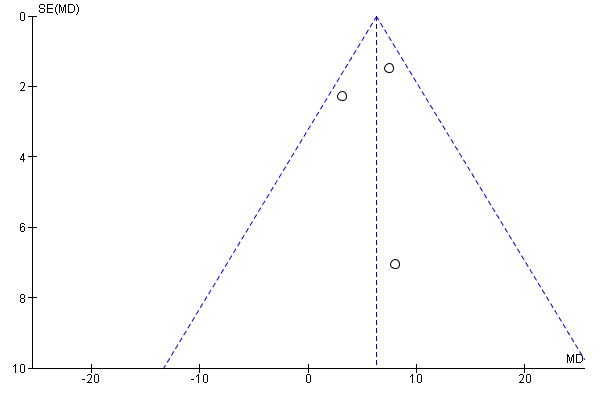


Figure S1. Funnel plot for metanalysis total Objective Structured Assessment of Technical Skills (OSATS) scores from RCT comparing VR training (VRT) and no additional training.


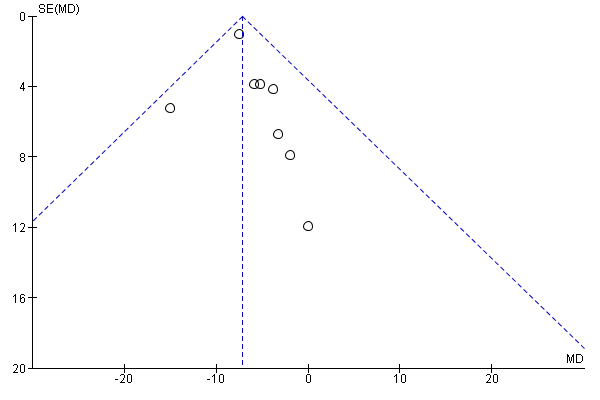


Figure S2. Funnel plot or metanalysis time to completion of task (minutes) from RCT comparing VR training (VRT) and no additional training (NAT).
